# Cytotoxic activity of peripheral blood mononuclear cells in patients with endometriosis: A cross-sectional study

**DOI:** 10.18502/ijrm.v20i8.11758

**Published:** 2022-09-06

**Authors:** Raden Muharam, Arleni Bustami, Indra Gusti Mansur, Teuku Zulkifli Jacoeb, Jerome Giustiniani, Valerie Schiavon, Armand Bensussan

**Affiliations:** ^1^Division of Reproductive Endocrinology and Infertility, Department of Obstetrics and Gynecology, Faculty of Medicine, Universitas Indonesia, Jakarta, Indonesia.; ^2^Master's Programme in Biomedical Sciences, Faculty of Medicine, Universitas Indonesia, Jakarta, Indonesia.; ^3^INSERM U976, Saint-Louis Hospital-Paris VII, Paris, France.; ^4^Mohammed VI-Polytechnic University, Ben Guerir, Morocco.

**Keywords:** CD28, CD160, Cytotoxic, Endometriosis, PBMC.

## Abstract

**Background:**

Endometriosis is believed to be associated with dysfunction of the lymphocyte population and cytotoxicity of natural killer (NK) cells, induced by the production of interleukin-2 (IL-2).

**Objective:**

This study aimed to investigate T lymphocytes and NK cell activity in the peripheral blood mononuclear cells (PBMCs) of women with endometriosis.

**Materials and Methods:**

PBMCs were obtained from the peripheral venous blood samples of 14 women with and without endometriosis (n = 7 for each group). Then, the PBMCs were co-cultured for 4 days and were treated with recombinant IL-2 for cytotoxic activity toward target cells (Daudi and K562 cells). The cytotoxicity activity was determined using the 51 chromium release assay before and after stimulation. Flow cytometry measurement was used to examine the expression of T lymphocytes and NK cells before and after being treated with IL-2.

**Results:**

The concentration of CD3+CD28+ (co-stimulatory) was significantly lower in the endometriosis group (65.62 ± 5.38) compared to in its counterpart (50.24 ± 4.22) (p = 0.04) before stimulation. However, no significant differences were observed in any other T lymphocytes and NK cells. It was also found that there was a significant increase of CD3-CD28+ after treatment with IL-2 only in the healthy control but not in women with endometriosis.

**Conclusion:**

Increased expression of CD160 and decreased CD28 play a role in inhibiting NK cell activation and T cell response in women with endometriosis.

## 1. Introduction

Endometriosis is a chronic inflammatory disorder characterized by the presence of endometrial-like tissue grows outside the uterine cavity (1). The etiopathogenesis of endometriosis is not yet well known, but one of the most prominent theories is of immunological dysfunction, causing defective immunosurveillance against aberrant autologous tissue in the peritoneal cavity, which facilitates endometriotic lesion growth in women with endometriosis (2).

The cytotoxicity of peripheral and peritoneal natural killer (NK) cells is decreased in women with endometriosis (3). However, the lower levels are due to a functional defect, not a proportion defect of the NK cells (4). One of the methods to overcome the reduced cytotoxicity is by stimulating the NK cells using stimulatory cytokine, i.e., interleukin-2 (IL-2). IL-2 stimulated NK cells which regulate the production of lymphokine-activated killer cells, indicating an increased potential against resistant tumor cells, including endometrial cells, both in-vitro and in women with endometriosis (5).

The cluster of differentiation 28 (CD28) is a co-stimulatory signal in T cells that modulates the function of both effector T cells and T-reg cells (6). CD28 is also a co-receptor and is involved in inducing T-cell activation. The function of CD28 on NK cells is yet unknown; however, previous studies reported that CD28 triggering in mice instigated not only NK cell proliferation but also cytotoxicity and cytokine secretion (7). Another study also demonstrated that activation of mouse IL-2-stimulated NK cells regulated the cytotoxic T-lymphocyte-associated protein 4 expression and CD28 elevation (8). These findings suggested that CD28 may be involved in the co-stimulatory pathway throughout the activation of T-cells. Additionally, as CD28 regulates the secretion of cytokines, CD28 might be a therapeutic target to regulate the production of T-cell cytokines.

CD160 is a unique NK cell receptor that behaves as an activating receptor on CD56 ${}^{\text{dim}}$ CD16 ${}^{+}$ NK cells, which mediates natural and antibody-dependent cellular cytotoxicity (9). CD160 can also promote NK cell cytotoxicity and interferon- γ production in vitro and in vivo. It is also associated with CD8 T lymphocytes with cytolytic effector activity. Human CD160 expression is upregulated on CD8 T cells that lose CD28 expression (10). However, the role of CD160-expressing T cells in women with endometriosis is poorly understood and should be investigated separately.

Given that current data related to cytotoxic activity associated with endometriosis are still limited, this study was designed to investigate T lymphocytes and their cytotoxicity markers, especially CD28 as costimulatory and CD160 as co-inhibitory, in the peripheral blood mononuclear cells (PBMCs) of women with endometriosis.

## 2. Materials and Methods

### Study design

In this cross-sectional study, PBMC samples of 14 women with and without endometriosis were collected between January and February 2011 using the consecutive sampling method. Samples were stored, stimulated, and examined at the Diagnostic and Research Center-Innovate, Faculty of Medicine, Universitas Indonesia (DIARC, FMUI) and INSERM U976, Saint Louise hospital, Paris.

### Subjects and sample collection

Participants were divided into 2 groups of cases and controls (n = 7/each) based on the inclusion criteria. These criteria were: 1) women of reproductive age; 2) having menstrual cycles; 3) positive for endometriosis based on histopathological and laparoscopic evidence (for the case group); 4) negative for endometriosis based on laparoscopy (for the control group); and 5) consent to participate in the study. Women who were pregnant, using hormonal contraception, using an intrauterine device for less than 3 months, or suffering from salpingitis or malignancy were excluded. Diagnostic operative laparoscopy was performed in a total of 7 reproductive-age women whose endometriosis was classified during laparoscopy based on the revised American Fertility Society Classification (11). None of the women consumed any hormonal drug within 3 months before peripheral blood collection. Women who suffered from any endocrine disorders were excluded. Before laparoscopy, 9 mL of peripheral venous blood was collected from the women with endometriosis by venipuncture in a heparinized Hank's buffer tube and delivered to the laboratory. In addition, peripheral venous blood was also obtained from the healthy volunteers.

### Isolation of PBMCs

PBMCs were obtained from the heparinized peripheral venous blood samples of the women with and without endometriosis by Ficoll-Hypague gradient centrifugation (Sigma-Aldrich, St. Louis Missouri, USA) at 1500 rpm for 30 min. PBMCs were collected from the interphase. Phosphate-buffered saline (Sigma-Aldrich, St. Louis, Missouri, USA) was used to wash the cells and followed by re-suspended in Roswell Park Memorial Institute (RPMI) 1640 medium (Gibco, Massachusetts, USA). The viability of the cells was determined by trypan blue staining (0.2% v/v, Thermo Fisher Scientific, Massachusetts, USA). Then, the isolated PBMCs were aliquoted in cryovials and stored at -80 C until used or cultured for cytotoxicity.

### Cells

Different human cell lines including Daudi (CCL-213) and K562 (human myelogenous leukemia cell line) cells (purchased from American Type Culture Collection, ATCC, Rockville, MD, USA) were used. RPMI 1640 supplemented with 10% fetal calf serum (FCS, Gibco, Massachusetts, USA), 2 mM of L-glutamine, 100 U/mL of penicillin, and 100 μg/mL of streptomycin (all from Sigma-Aldrich, St. Louis, Missouri, USA) was used for all cells culture. The cells were maintained at 37 C under 5% humidified CO ${}_{2}$ atmosphere.

### Immuno-labelling of leukocytes and flow cytometry analysis

Cryopreserved PBMCs were thawed in a 37 C water bath as quickly as possible and were washed twice with RPMI 1640 medium to remove the dimethyl sulfoxide. The samples were used for flow cytometry analysis. The PBMCs were immunolabelled using antibodies against the following surface proteins: CD3, CD56, CD28, and CD160 (BD Biosciences, Franklin Lake, USA) according to the manufacturer's instructions. Flow cytometry was carried out using Fluorescence Activated Cell Sorting flow cytometers (Beckman Coulter FC50, Life Sciences, Indianapolis, USA). Data acquisition was carried out using Cell Quest software (BD Biosciences, Franklin Lake, USA).

### Cytotoxic activity of NK cells from PBMCs

PBMCs containing NK cells were placed into a 4 well cell culture plate at a concentration of 200,000 cells per well. Recombinant IL-2 was also added to all wells at a concentration of 100 U/mL for NK cell viability. PBMCs were co-cultured for 4 days (96 hr) in a humid environment at 37 C under 5% CO ${}_{2}$ . After 96 hr, lymphokine-activated killer cells were harvested, counted, and evaluated for cytotoxic activity toward Daudi and K562 cell lines (ATCC, Virginia, USA). The cytotoxicity of the NK cells mediated by antibody immunotherapy was also determined using chromium (^51^Cr) release assay. Cell targets (Daudi cells and K562 cell lines) were labeled with 100 μCi of ^51^Cr mCi/mg (Perkin Elmer, Massachusetts, USA) for 4 hr at 37 C under 5% CO ${}_{2}$ . Cell targets were washed 3 times with RPMI 1640 at 4 C and resuspended at a concentration of 5 x 10^4^/mL in RPMI 1640 with 10% FBS. The effector cells were resuspended in a complete medium and the cells were counted with trypan blue to assess the cells' viability. Cells were co-incubated at a ratio of 50:1, 25:1, and 5:1 between effector to target (E: T) cells. In each well, the final concentrations were 100 μL of effector cells and 100 μL of target cells. After 4 hr of incubation, 100 μL was removed from each well, and radioactivity was counted γ counter to determine ^51^Cr release. Radioactivity was measured by Trilux Micro Beta Automatic Gamma Counter (PerkinElmer, Massachusetts, USA). The percentage of cytotoxic activity was calculated using the following formula: 


$$Lysis\left(\%\right)=\frac{Experimental{Cr}^{51}release-spontaneous{Cr}^{51}release}{Maximum{Cr}^{51}release-spontaneous{Cr}^{51}release}x100$$

### Ethical considerations

All women gave written informed consent. The study protocol was approved by The Ethics Review Committee of the Faculty of Medicine, Universitas Indonesia (No. 390/PT02.FK/ETIK/2009).

### Statistical analysis

Statistical analyses were performed using the Statistical Package for Social Sciences software version 22.0 (SPSS Inc., Chicago, IL, USA). A normality test was performed using Kolmogorov-Smirnov test. Depending on normality distribution, independent *t* test or the Mann-Whitney test was used. The paired *t* test or Wilcoxon test was used to examine the pre- and post-treatment. A value of p less than 0.05 was considered significant.

## 3. Results

The demographic characteristics of the groups are presented in table I. The mean age of the women with endometriosis group was not different from that of the women without endometriosis group. No difference in waist circumference or body mass index was observed between the 2 groups.

**Figure 1:**
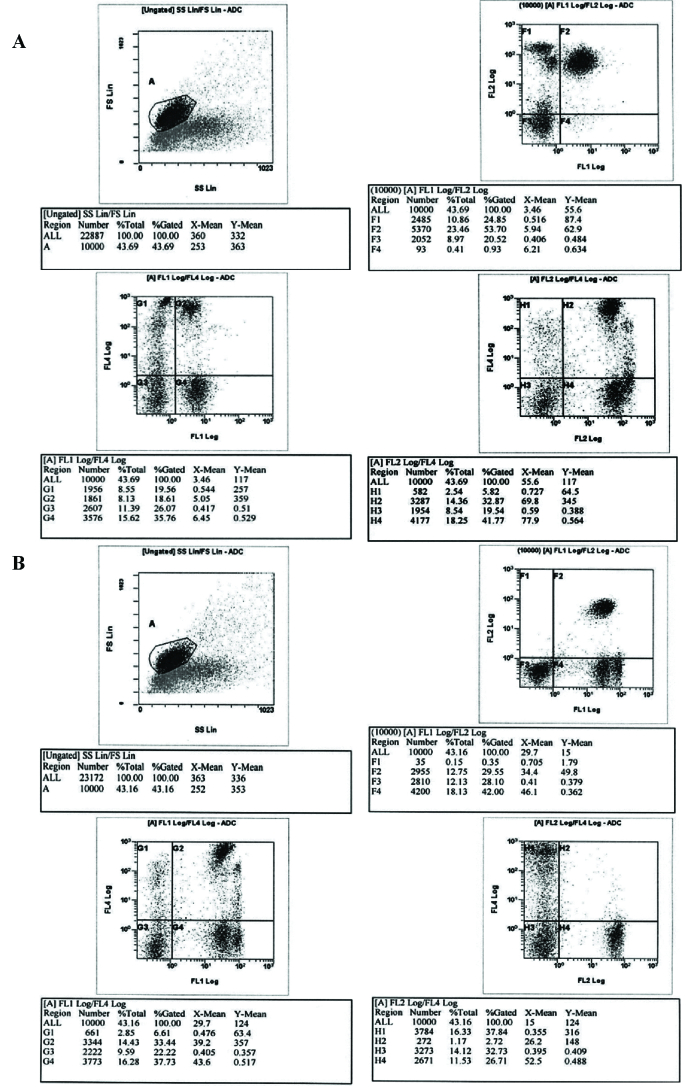
Representative flow cytometry data of CD28 ${}^{+}$ (a) and CD160 ${}^{+}$ (b). Mononuclear cells were isolated from the peripheral blood of women with and without endometriosis and were stained. The lymphocytes were firstly gated on an FSC/SSC scatter graph, and then were identified as double-positive cells (CD3 ${}^{+}$ CD28 ${}^{+}$ and CD56 ${}^{+}$ CD160 ${}^{+}$ ).

To evaluate the concentrations of CD28 and CD160, flow cytometric analysis was carried out (Figure 1). The results were based on analysis of at least 100,000 cells and are shown as the percentage of positively labelled cells.

The concentration of CD3+CD28+ (co-stimulatory) was significantly lower in the endometriosis group (50.24 ± 4.22) compared to in its counterpart (65.62 ± 5.38) (p = 0.04) before stimulation. However, no differences in any other lymphocyte subpopulations were discovered between the 2 groups before the culturing (Table II).

**Figure 2:**
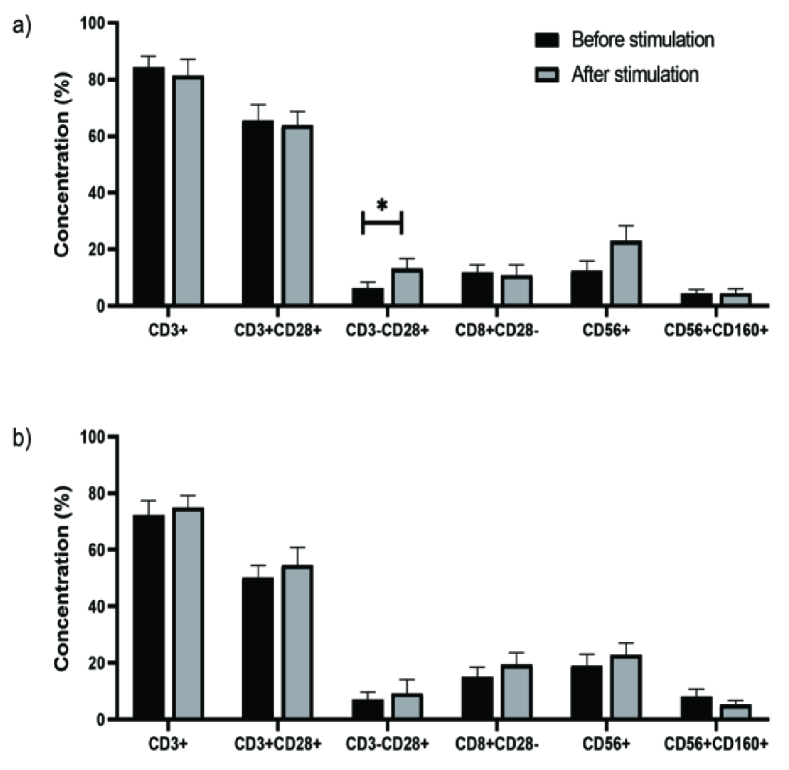
T lymphocytes and NK cell concentration with IL-2 stimulation.(a) The concentration of the lymphocyte population in the control group. (b) The concentrations of the lymphocyte subpopulation in the endometriosis group. Column chart box = 95 ${}^{\text{th}}$ percentiles, Whiskers: Extend to the extreme values. Differences before and after IL-2 stimulation were analyzed by paired *t* test. *Significant at the level of 5%.

After culturing with IL-2 for 4 days, the concentrations of NK cells in the control group did not change (Figure 2a). No differences were observed in the lymphocyte subpopulation in the endometriosis group after being stimulated with IL-2 (Figure 2b).

**Figure 3:**
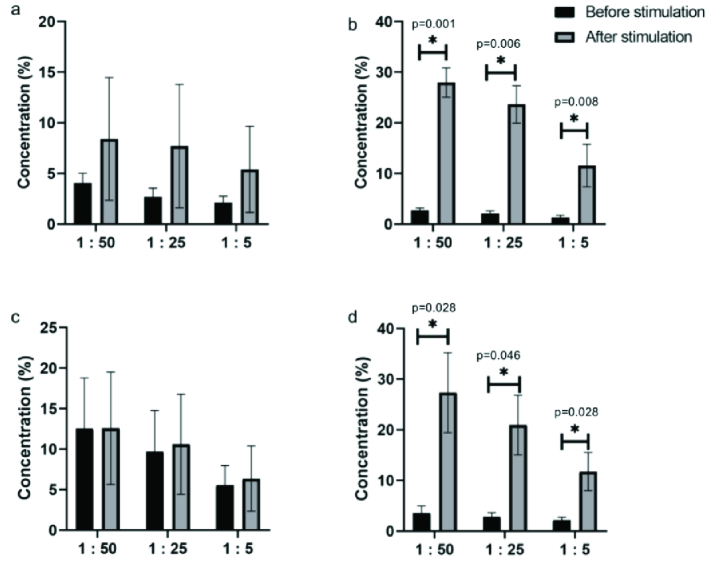
Cytotoxic activity of T lymphocytes and NK cells with ^51^Cr assay. Each graph shows the expression changes of the indicated T lymphocytes and NK cells during the 4 hr of IL-2 treatment. The data represent the median values for each indicated treatment. a) Daudi cells in the control group, b) Daudi cells in the endometriosis group, c) K562 cells in the control group, and d) K562 cells in the endometriosis group. Differences before and after IL-2 stimulation were analyzed by Wilcoxon signed-rank test. *Significant at the level of 5%.

We also evaluated the cytotoxicity of effector cells towards target cells. Pre-treatment with IL-2 for 4 hr enhanced the spontaneous cytotoxicity of human PBMCs against a variety of target cells, including Daudi and K562 (Figure 3). The significant enhancement of lymphocyte T and NK cell cytotoxicity was observed in all doses of IL-2 against both Daudi and K562 cells in the endometriosis group.

**Table 1:** Demographic characteristics of the subjects


**Variables**	**Control group**	**Endometriosis group**	**P-value**
**Age (yr)**	33.71 ± 2.14	30.57 ± 2.33	0.34
**Waist circumference (cm)**	99.43 ± 2.76	93.00 ± 3.12	0.15
**Weight (kg)**	60.43 ± 3.85	52.29 ± 1.60	0.08*
**Height (cm)**	156.86 ± 2.44	154.86 ± 1.74	0.52
**Body mass index (kg/m^2^)**	24.43 ± 1.09	21.83 ± 0.77	0.08*
Data shown as Mean ± Standard deviation. *Significant at the level of 5%. Independent *t* test			

**Table 2:** T lymphocytes and NK cells of PBMCs in the women


**Variables**	**Control group**	**Endometriosis group**	**P-value**
**CD3^+^**	84.29 ± 3.99	72.23 ± 5.24	0.09
**CD3^+^CD28^+^**	65.62 ± 5.38	50.24 ± 4.22	0.04*
**CD3^–^CD28^+^**	6.33 ± 2.05	7.01 ± 2.80	0.85
**CD8^+^CD28^–^**	11.89 ± 2.65	15.16 ± 3.26	0.45
**CD56+**	12.50 ± 3.29	19.08 ± 4.10	0.24
**CD56+ CD160+**	4.47 ± 1.42	8.22 ± 2.56	0.22
Data shown as Mean ± Standard deviation. *Significant at the level of 5%. Independent *t* test			

## 4. Discussion

The changes in immune reactions which are normally responsible for detecting and removing abnormal growing tissue are likely to play a crucial role in the endometriosis development (6). Some immune cells including monocytes/macrophages and NK cells change in quantity and quality in the peritoneal environment of women with endometriosis. The function of NK cells is triggered by the interactions of the various combinations of activating or inhibiting surface molecules with the target cells (12). Reduction in NK cells' cytotoxic function has been observed in women with endometriosis, especially in the peritoneal fluid, allowing the survival and implantation of the endometrial cells outside the uterine cavity.

Activation of effector T lymphocytes is mediated by costimulatory molecules, including CD28, which deliver signals essential for the development and homeostasis of suppressive regulatory T cells. CD28 participates in an intracellular signaling pathway in T cells (13). CD28, acting through phosphatidylinositol 3'-kinase, is needed for T cells to increase their glycolytic response to activation (14). In this study, we found that the expression of CD3 ${}^{+}$ CD28 ${}^{+}$ was significantly lower in the peripheral blood of women with endometriosis than in the control group women. However, a higher frequency of CD8 ${}^{+}$ CD28 ${}^{-}$ (T suppressor cells) was observed in the women with endometriosis. These results are in line with the previous study that found that the frequency of T suppressor cells was increased in endometriosis (15). It may be possible that the inhibition of the immune response in the endometriosis microenvironment consists of several inhibitory mechanisms, including decreasing of costimulatory factors, which is revealed in our study.

CD160 is expressed on NK cells, CD8 ${}^{+}$ T cells, and a small subset of CD4 ${}^{+}$ T cells, and it promotes the cytotoxicity of NK cells and cytokine production (9). In this study, we found no significant difference in the expression of CD160 in the peripheral blood obtained from women with vs. without endometriosis. The expression of CD160 was elevated in the women with endometriosis compared to the control group women. In our previous study, we found that the expression of CD4 ${}^{+}$ was lower in the peripheral blood of women with endometriosis compared to their counterparts (16). The high expression of CD160 is believed to be one of the factors that plays a role in decreasing CD4 ${}^{+}$ expression in women with endometriosis because the bond between CD160 and its ligand inhibits the activation of CD4 ${}^{+}$ T cells (15).

NK cell activity is also influenced by soluble immunostimulatory cytokines such as IL-2 (17). It was found that the expression of CD28 ${}^{+}$ was higher in the women with endometriosis after being stimulated with IL-2. It has been shown that CD28 promotes T cell proliferation due to the accumulation of IL-2 (18). Thus, CD28 can deliver biochemical signals to initiate and maintain T cell responses. In this study, it was also found that there was no change in CD160 expression after being treated with IL-2 in both the endometriosis and control groups. The expression of CD160 was downregulated in the endometriosis group after stimulation. CD160 does not proliferate in response to IL-2 stimulation, which might be due to the high potential of its cytotoxic activity (19). Furthermore, a previous study using CD160 mice demonstrated that CD160 is important for interferon- γ production mediated by NK cells, and the sufficiency of CD160 ${}^{+}$ expression may be necessary for controlling tumor development (19).

## 5. Conclusion

In conclusion, this study, combined with a previous one (16), demonstrated that the increased expression of CD160 and decreased expression of CD28 may play a role in inhibiting NK cell activation and T cell response in endometriosis.

##  Conflict of Interest

The authors declare that there is no conflict of interest.
